# Supplementary material for: Global coastal attenuation of wind-waves observed with radar altimetry
Source: Nat Commun. 2021 Jun 21;12:3812. doi: 10.1038/s41467-021-23982-4 (PMC8217570; doi:10.1038/s41467-021-23982-4)
Supplement: Supplementary file 1 — Supplementary Information [file 41467_2021_23982_MOESM1_ESM.pdf]

Supplementary figures

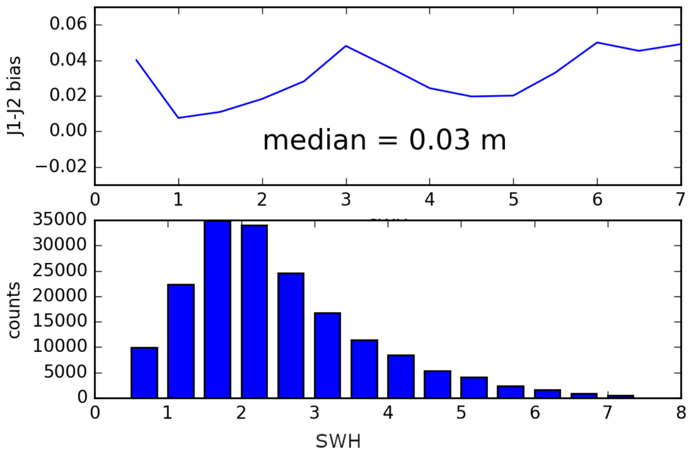

Supplementary Figure 1: Difference of SWH between Jason-1 and Jason-2 measurements as function of the average SWH in bins of 0.5 m and number of available measurements

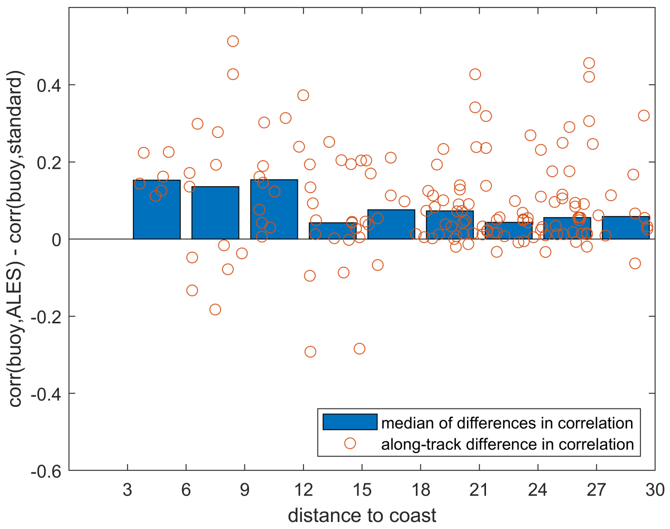

Supplementary Figure 2: Difference in correlation between altimetry and buoy time series when considering the ALES dataset or the standard GDR dataset, with respect to the distance to the coast of the altimetry point location. Positive numbers indicates a higher correlation using ALES data. Values at the single along-track locations are shown in circles, while a median of the differences is shown in 3-km-long bins.

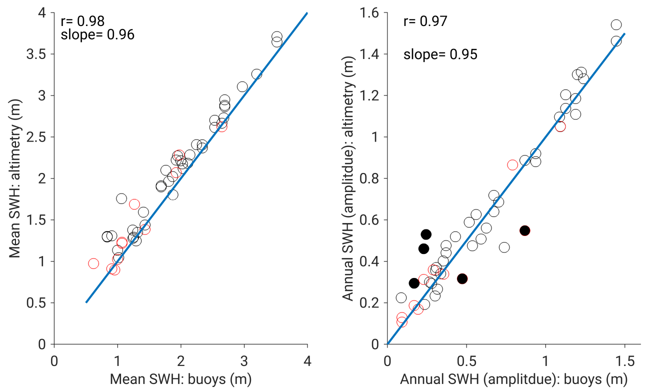

Supplementary Figure 3: Left: Scatter plot of mean SWH from buoys and altimetry. Right: Scatter plot of the amplitude of the annual cycle from buoys and altimetry. Coastal buoys are highlighted in red. Filled circles correspond to couples in which the difference of the estimated amplitudes is statistically significant. Value of Pearson's correlation coefficient (r) and slope of the linear regresssion are reported on the top left of each plot.

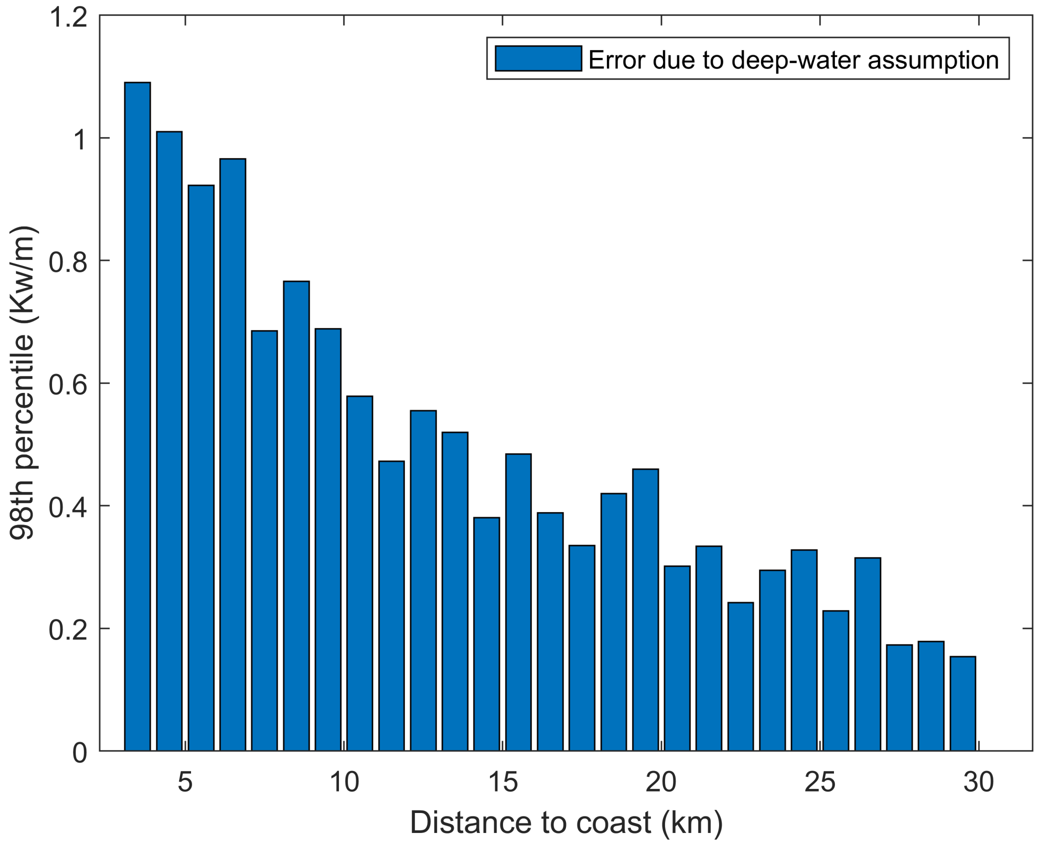

Supplementary Figure 4: 98th percentile of the differences between the computation of the average energy flux using the deep water assumption and the approximate solution considering shallow and intermediate waters.

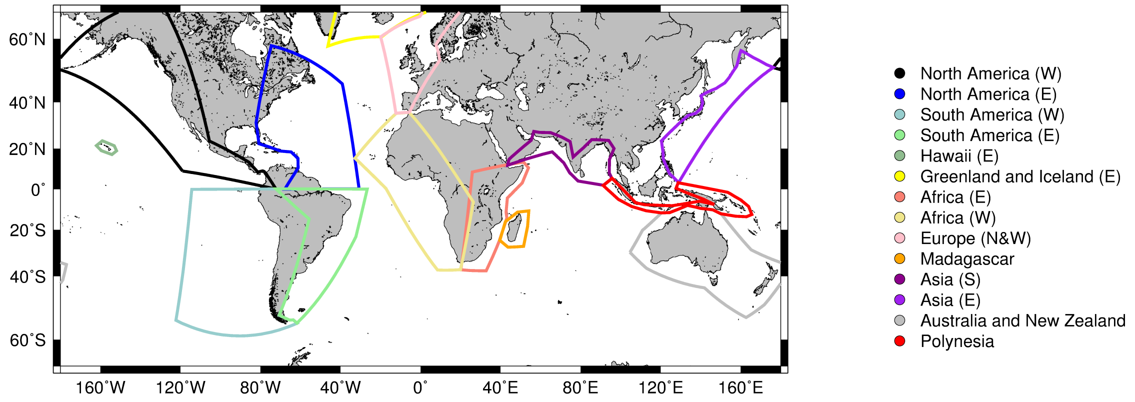

Supplementary Figure 5: Subdivision of the global coastal ocean adopted in this study.
